# Unveiling ceramide dynamics: Shedding light on healthy aging in growth hormone‐releasing hormone knockout mice

**DOI:** 10.1111/acel.14226

**Published:** 2024-05-29

**Authors:** Alexander Tate Lasher, Liping Wang, Jooyoung Hyun, Scott A. Summers, Liou Y. Sun

**Affiliations:** ^1^ Department of Biology University of Alabama at Birmingham Birmingham Alabama USA; ^2^ Department of Nutrition and Integrative Physiology University of Utah Salt Lake City Utah USA

**Keywords:** aging, ceramide, GHRH, lifespan, lipidomics, longevity, metabolic

## Abstract

Dysregulation of growth hormone (GH) signaling consistently leads to increased lifespan in laboratory rodents, yet the precise mechanisms driving this extension remain unclear. Understanding the molecular underpinnings of the beneficial effects associated with GH deficiency could unveil novel therapeutic targets for promoting healthy aging and longevity. In our pursuit of identifying metabolites implicated in aging, we conducted an unbiased lipidomic analysis of serum samples from growth hormone‐releasing hormone knockout (GHRH‐KO) female mice and their littermate controls. Employing a targeted lipidomic approach, we specifically investigated ceramide levels in GHRH‐KO mice, a well‐established model of enhanced longevity. While younger GHRH‐KO mice did not exhibit notable differences in serum lipids, older counterparts demonstrated significant reductions in over one‐third of the evaluated lipids. In employing the same analysis in liver tissue, GHRH‐KO mice showed pronounced downregulation of numerous ceramides and hexosylceramides, which have been shown to elicit many of the tissue defects that accompany aging (e.g., insulin resistance, oxidative stress, and cell death). Additionally, gene expression analysis in the liver tissue of adult GHRH‐KO mice identified substantial decreases in several ceramide synthesis genes, indicating that these alterations are, at least in part, attributed to GHRH‐KO‐induced transcriptional changes. These findings provide the first evidence of disrupted ceramide metabolism in a long‐lived mammal. This study sheds light on the intricate connections between GH deficiency, ceramide levels, and the molecular mechanisms influencing lifespan extension.

## Graphical abstract

Dysregulation of GH signaling extends lifespan in rodents, but the mechanisms remain unclear. Our lipidomic analysis of GHRH‐KO mice reveals reduced ceramide levels in serum and liver tissue, suggesting altered metabolism in long‐lived mammals. This sheds light on the link between GH deficiency, ceramide levels, and lifespan extension.

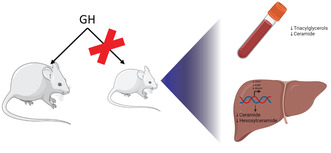

**Abbreviations**
*Acsm5*
: acyl‐CoA synthetase medium‐chain family member 5
*Actb*
: actin, beta
*Adipor1‐2*
: adiponectin receptor (1‐2)
*Asah1‐2*
: N‐acylsphingosine amidohydrolase (1‐2)
CE: cholesterol esters
Cer: ceramides
*Cers1‐6*
: ceramide synthase (1‐6)
*Degs1‐2*
: delta 4‐desaturase, sphingolipid (1‐2)
DG: diacylglycerols
*Fabp1*
: fatty acid binding protein 1
GH: growth hormone
GHRH: growth hormone‐releasing hormone
GHRH‐KO: growth hormone‐releasing hormone knockout
HexCer: hexosylceramides
*Igf1*
: insulin‐like growth factor 1
*Kdsr*
: 3‐ketodihydrosphingosine reductase
PC: phosphatidylcholines
SM: sphingomyelin
*Sphk2*
: sphingosine kinase 2
*Sptlc1‐3*
: serine palmitoyltransferase, long chain base subunit (1‐3)
TG: triacylglycerols
WT: wild‐type

Aging can briefly be defined as the time‐dependent loss of physiological function in an organism ultimately resulting in death (López‐Otín et al., 2013). Unsurprisingly, this broad loss of function positions advanced age as a critical risk factor for the development of chronic conditions such as Type 2 diabetes or neurodegeneration (Franceschi et al., 2018). The detriments of old age coupled with a rapidly aging population (Partridge et al., 2018) necessitate close study of the biology of aging to elucidate causal mechanisms for these detriments and uncover potential targets for prolonging lifespan. This positions long‐lived animal models as valuable resources for better understanding mammalian longevity, as differences in their physiology likely contribute to their extended lifespan.

Disruption of growth hormone (GH) signaling is among the most reproducible methods of lifespan extension in laboratory rodents. Hypopituitary dwarf mice, deficient in GH and other pituitary‐derived hormones, GH‐receptor knockout mice, and GH‐releasing hormone knockout (GHRH‐KO) mice all display significantly extended lifespan compared to mice with a functional GH axis (Brown‐Borg et al., 1996; Coschigano et al., 2000, 2003; Flurkey et al., 2001; Sun et al., 2013). That this has been repeatedly observed in both male and female mice on several genetic backgrounds across multiple laboratories indicates that this pathway is critically involved in the regulation of mammalian lifespan. Among the notable features of GH‐disrupted mice, besides their longer lives, is their altered lipid homeostasis. We have previously shown that GHRH‐KO mice have disproportionately greater fat mass (after accounting for body weight differences) and preferentially oxidize lipids relative to carbohydrates, like other GH‐deficient mouse models (Hill et al., 2016; Icyuz et al., 2020; Lasher & Sun, 2023; Longo et al., 2010; Westbrook et al., 2009). These differences in total body lipid metabolism warrant investigation into how lipid signaling is differently regulated in long‐lived GH‐deficient mice.

Sphingolipids, particularly ceramides, have been shown to play important roles in regulating the lifespan across several model organisms. Genetic and pharmacological interventions lowering sphingolipid synthesis in the budding yeast *Saccharomyces cerevisiae* have been shown to increase the replicative lifespan (Huang et al., 2012; Swinnen et al., 2014). In *Caenorhabditis elegans* and *Drosophila melanogaster*, reduced expression of genes involved in ceramide metabolism confers extended lifespan over controls (Mosbech et al., 2013; Tedesco et al., 2008; Yang et al., 2010). While no lifespan analysis has been carried out in mammals with interrupted sphingolipid metabolism, it has been reported that sphingolipid accumulation and sphingolipid catabolic activity both increase with age in rodents (Giusto et al., 1992; Lightle et al., 2000; Sacket et al., 2009). Further, ceramide accumulation has been associated with advanced age in humans (Ishikawa et al., 2014; Mielke et al., 2015). Here, we present the first evidence that sphingolipids and ceramide abundance are reduced in long‐lived GHRH‐KO mice, implicating these lipids as an important lifespan regulator.

We carried out a targeted lipidomic analysis to assess the concentration of sphingolipids and related lipids in serum from GHRH‐KO and WT female mice. Principal component analysis revealed no discernable differences between the younger (3–5 months old) mice; however, a distinct grouping was observed between the older (10–15 months old) GHRH‐KO and WT mice (Figure 1a). We detected 46 significantly less abundant lipids in these older GHRH‐KO mice compared to their age‐matched WT controls (Figure 1b), while no significant differences were observed between the genotypes of younger mice (Figure 1c). Among the less abundant lipids in the older GHRH‐KO mice were diacylglycerols (DG), triacylglycerols (TG), ceramides (Cer), phosphatidylcholines (PC), hexosylceramides (HexCer), cholesterol esters (CE), and sphingomyelin (SM). Heat map visualization of these lipids for all groups analyzed revealed no appreciable patterning for these lipids in the younger mice (Figure 1d). These data suggest a shift in the circulating lipidome in this model of extreme longevity, with GHRH‐KO females accumulating fewer lipids in systemic circulation through adulthood.

**FIGURE 1:**
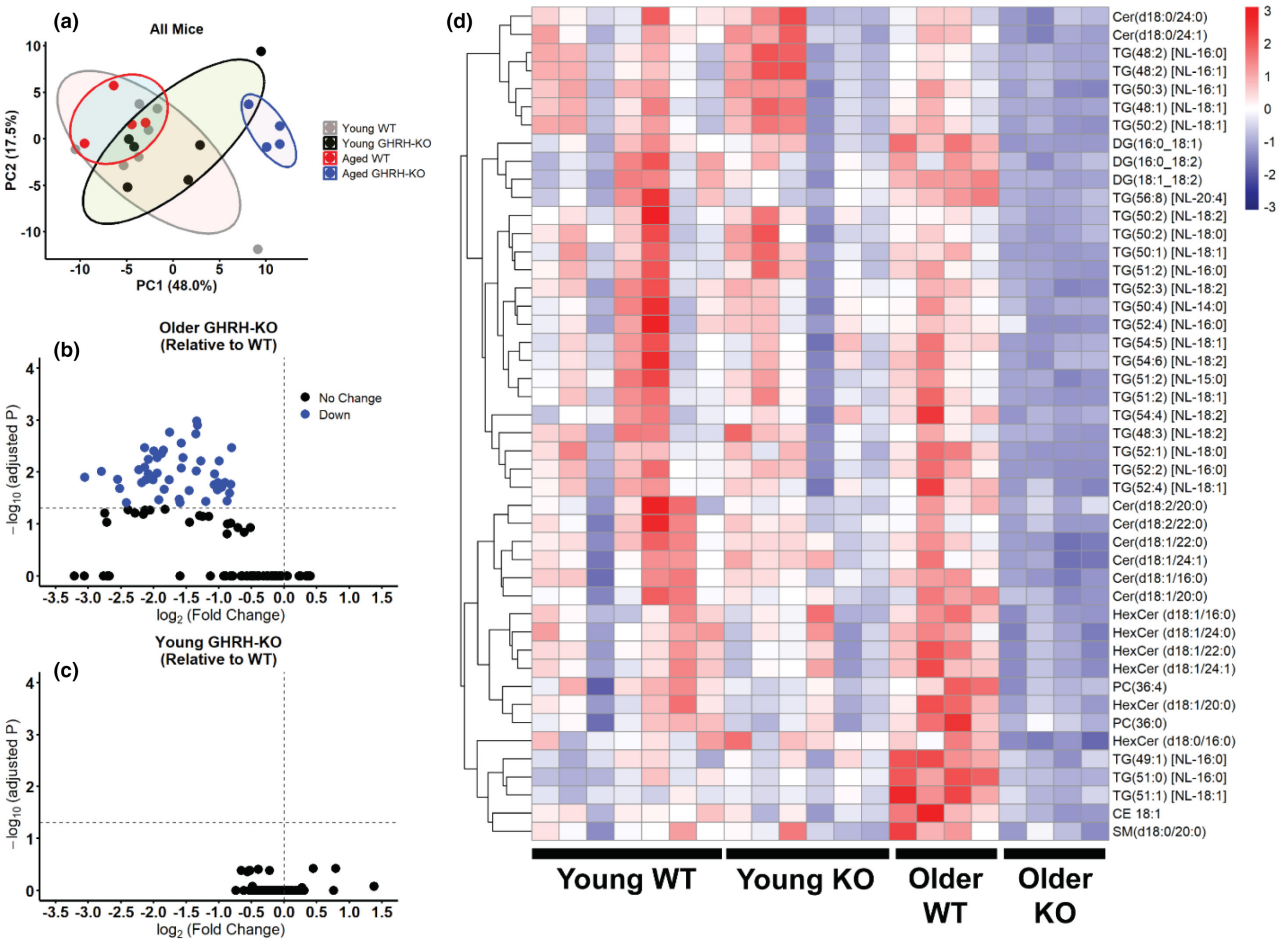
Reduced circulating lipids in GHRH‐KO adult females. Plots of principal component (PC) analysis for serum lipid abundance in young (3–5 months old) and aged (10–15 months old) WT and GHRH‐KO mice (a). Volcano plots of lipids in 10‐ to 15‐month‐old (b) and 3‐ to 5‐month‐old (c) mice. Black points indicate no significant difference in lipid abundance, blue points indicate significantly (adjusted *p* < 0.05) decreased lipid abundance. Heat map of the significantly (adjusted *p* < 0.05) different lipids in the 10‐ to 15‐month‐old GHRH‐KO mice relative to WT mice represented for all mice assessed, with the color scale representing *z*‐score (d). Statistical significance determined by two‐tailed Student's *t* test followed by sequential goodness of fit correction for multiple testing. *N* = 6–7 per group (3‐ to 5‐month‐old mice) or *N* = 4 (10‐ to 15‐month‐old mice).

Previous work has shown that GH‐deficient male Ames dwarf mice display notable alterations in tissue lipid profiles, defined by increased cardiolipin and reduced ceramide abundance at 6 months old (Darcy et al., 2020). To further investigate the changes in the lipid profile of our long‐lived GHRH‐KO mice, we carried out the same targeted lipidomic analysis as in serum in the liver tissue of 6‐ to 8‐month‐old GHRH‐KO and WT females, an age representative of mature adulthood evidenced by minimal changes in female bodyweight during this time (Icyuz et al., 2020). We selected the liver as this is a major site of insulin‐like growth factor 1 (IGF‐1) production, a key mediator of GH's effects (Aguiar‐Oliveira & Bartke, 2019), and because GHRH‐KO mice display marked reduction in hepatic *Igf1* expression (Figure S1A). Principal component analysis revealed separation between the two groups, although to a lesser extent than what was observed in 10‐ to 15‐month‐old mice (Figure 2a). We detected 19 lipids in GHRH‐KO livers with significantly different abundance compared to WT livers (Figure 2b). Of these 19 lipids, 12 were either ceramides or hexosylceramides, and their concentration was generally significantly lower in GHRH‐KO mice relative to WT mice (Figure 2c,d). To determine if these changes in liver ceramides were due to transcriptional changes, we quantified mRNA transcript abundance by RT‐qPCR in a separate cohort of 7‐month‐old GHRH‐KO and WT female livers. Expression of several genes known to be involved in ceramide biosynthesis (Meikle & Summers, 2017) was significantly reduced in GHRH‐KO mice (Figure 2e). We also observed reduced expression of *Adipor1* and *Adipor2*, known to possess ceramidase activity (Holland et al., 2011), and the acid ceramidase *Asah1*, however, only *Adipor1* reached our criteria for statistical significance (Figure S1B). We also observed a trend (although not statistically significant) for reduced expression of *Sphk2* (Figure S1C). These data, in conjunction with the data presented in Figure 1, indicate dampened ceramide metabolism during adulthood in this model of extended longevity.

**FIGURE 2:**
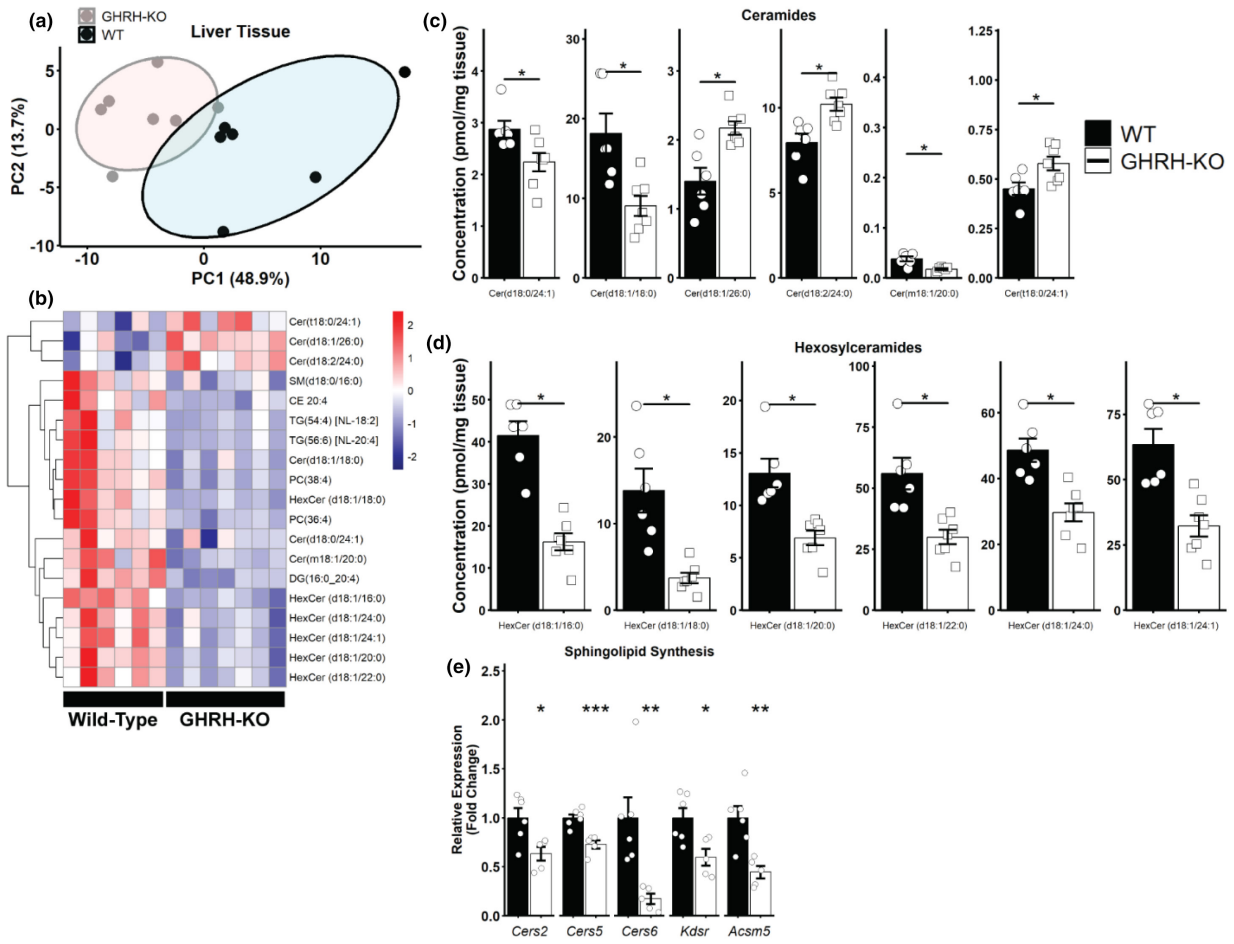
Reduced ceramide abundance in GHRH‐KO mouse liver. PC analysis plot for liver lipid abundance in 6‐ to 8‐month‐old GHRH‐KO and WT female livers (a). Heat map of the significantly (adjusted *p* < 0.05) different lipids in the GHRH‐KO livers relative to WT mice, with the color scale representing *z*‐score (b). Significantly (adjusted *p* < 0.05) different ceramide (c) and hexosylceramide (d) concentration in GHRH‐KO and WT livers. Relative hepatic mRNA abundance of genes involved in ceramide synthesis, presented as fold change relative to WT mice (e). *p < 0.05; **p < 0.01; ***p < 0.001; statistical significance determined by two‐tailed Student's *t* test (e) with sequential goodness of fit correction for multiple testing (a–d). *N* = 6–7 (a–d) or *N* = 5–6 per group (e). Bars show mean ± SEM with points representing individual mice. *Actb* was used as an endogenous control for mRNA expression.

Among the notable features of GH‐deficient mice is that they display reduced rates of aging during adulthood (Koopman et al., 2016; Sun et al., 2017), indicating that these mice have both extended health spans and lifespans. This indicates that the aging process itself is slowed in these animals. Consistent with this notion, the incidence of chronic age‐associated conditions such as cancer, cognitive decline, and insulin resistance (Ikeno et al., 2003; Kinney, Coschigano, et al., 2001; Kinney, Meliska, et al., 2001; Podlutsky et al., 2017; Wiesenborn et al., 2014; Zhang et al., 2020) is reduced in GH‐deficient rodents. A growing body of literature implicates ceramide accumulation in the development of these same conditions (Holland et al., 2007; Li et al., 2023; Ordóñez‐Gutiérrez et al., 2018). It is noteworthy that our older GHRH‐KO mice displayed significant reductions in serum TGs in addition to ceramides. Reductions in blood TGs have previously been reported in GH‐deficient mice (Gesing et al., 2014; Hill et al., 2016), and inhibition of ceramide synthesis has been shown to dramatically reduce TG accumulation in blood (Holland et al., 2007). In conjunction with the lower expression of ceramide synthesis genes in GHRH‐KO mice, we pose that transcriptional changes conferred by GHRH deletion result in these lipidomic changes. These reports together with our data presented here may begin to provide a novel mechanism for the improved health span of GH‐deficient mice where less ceramide accumulates with age, delaying physiological decline in these mice. Recently, lowering ceramide synthesis in later adulthood has been shown to improve measures of fitness in aged mice (Lima et al., 2023) supporting the hypothesis that ceramide accumulation contributes to age‐related decline.

In brief, we report noteworthy differences in circulating sphingolipids between adult GHRH‐KO and WT female mice. We go on to show that these differences are also present in the liver with ceramides being particularly less abundant in GHRH‐KO mice, and that reduced expression of key ceramide synthesis genes likely contributes to this. To our knowledge, our study is the first to demonstrate that sphingolipid metabolism is disrupted in a long‐lived rodent model and may begin to provide a causal link between the altered lipid metabolism and extreme longevity in GH‐deficient mice.

## 1 METHODS SUMMARY

The mice (Icyuz et al., 2020) and sphingolipid assessment protocol (Chaurasia et al., 2019; Poss et al., 2022) have been described. Gene expression from liver RNA was assessed by qPCR using the 2^−ddCt^ calculation. Method details are provided in the Appendix S1.

## AUTHOR CONTRIBUTIONS

LYS and SAS conceived the study, secured funding, and managed overall direction. LYS, SAS, and ATL designed experiments. LW and LH collected data. ATL, LW, and JH analyzed data. ATL took the lead in writing the manuscript. All authors contributed edits and provided critical feedback that helped shape the final manuscript.

## FUNDING INFORMATION

The authors received research support from the National Institutes of Health AG082327, AG057734 to LYS; CA272529, DK115824, DK116888, DK116450, and DK130296 to SAS.

## CONFLICT OF INTEREST STATEMENT

SAS and LPW are shareholders of Centaurus Therapeutics, Inc. All other authors declare no conflict of interest.

## Supporting information


Appendix S1.

## Data Availability

All of the data supporting the findings of this study are available from the corresponding author upon reasonable request.
