# Supplementary material for: Unveiling ceramide dynamics: Shedding light on healthy aging in growth hormone‐releasing hormone knockout mice
Source: Aging Cell. 2024 May 29;23(8):e14226. doi: 10.1111/acel.14226 (PMC11320351; doi:10.1111/acel.14226)
Supplement: Supplementary file 1 — Appendix S1. [file ACEL-23-e14226-s001.docx]

Supplemental Table 1: Primer sequences used for RT-qPCR

| **Symbol** | **Forward (5’ 🡪 3’)** | **Reverse (5’ 🡪 3’)** |
| --- | --- | --- |
| *Actb* | CCAGTTGGTAACAATGCCATGT | GGCTGTATTCCCCTCCATCG |
| *Cers1* | CTGTTCTACTTGGCCTGTTG | GTGGAGGTAGACCTTTTGTCAC |
| *Cers2* | GTGGAGGTAGACCTTTTGTCAC | CTGGCTTCTCGGAACTTTTT |
| *Cers3* | GCGCTTTGGGAAGAAGAATGTTT | TGGGAAGCTTTCACGAAGACA |
| *Cers4* | ACCTCCAGCATGTCGTTCAG | AGGGGCAAGGCCACAAAT |
| *Cers5* | CCAATGCTGGTTTCGCCATC | GAACCAAGGCATCGACCAGA |
| *Cers6* | GTTCGGAGCATTCAACGCTG | CTGAGTCGTGAAGACAGAGG |
| *Degs1* | ATGGGCCTCTGAACTTGCTC | CGATCTTCCTCACCATGGGC |
| *Degs2* | TGAAGGGCCACGAGACTTAC | GTGCAATCTTCCGCACCAGT |
| *Kdsr* | AAAACAAAACGAAGCCCCTGG | ACAGCATGTACCCATCTGAGC |
| *Acsm5* | TGACACTGGATGGGTGAAAG | GGGAACCTGCAGAGAGTATTTAG |
| *Sphk2* | CACGGCGAGTTTGGTTCCTA | CTTCTGGCTTTGGGCGTAGT |
| *Asah1* | CAGGACTGTTCAGTCTTTCAC | GAGTGATAAACCCTACCCACT |
| *Asah2* | GCAAAGCGAACCTTCTCCAC | AGGGCCACTGTGATGACTGT |
| *Adipor1* | TCTGCCTCAGTTTCTCCTGGCT | GTAATAGAGCCAGGGAACGAAGC |
| *Adipor2* | TCTTCCACACGGTGTACTGCCA | GGTAGATGAAGCAAGGTTGTGGG |
| *Sptlc1* | GAGGAGTCACCGAGCACTATGG | GCTGATGGTCAACCACGAAGGA |
| *Sptlc2* | CCAGACTGTCAGGAGCAACCAT | CTTCTTGTCCGAGGCTGACCAT |
| *Sptlc3* | GCCCGCTAAAGTGTCTGCTT | TCCCGAGTATGTGCAGAGGA |
| *Fabp1* | CTTCTCCGGCAAGTACCAAT | CCTTGATGTCCTTCCCTTTCT |
| *Igf1* | CATAGTACCCACTCTGACCTGCTGTG | CGCCAGGTAGAAGAGGTGTGAAGAC |

Supplemental Figure S1


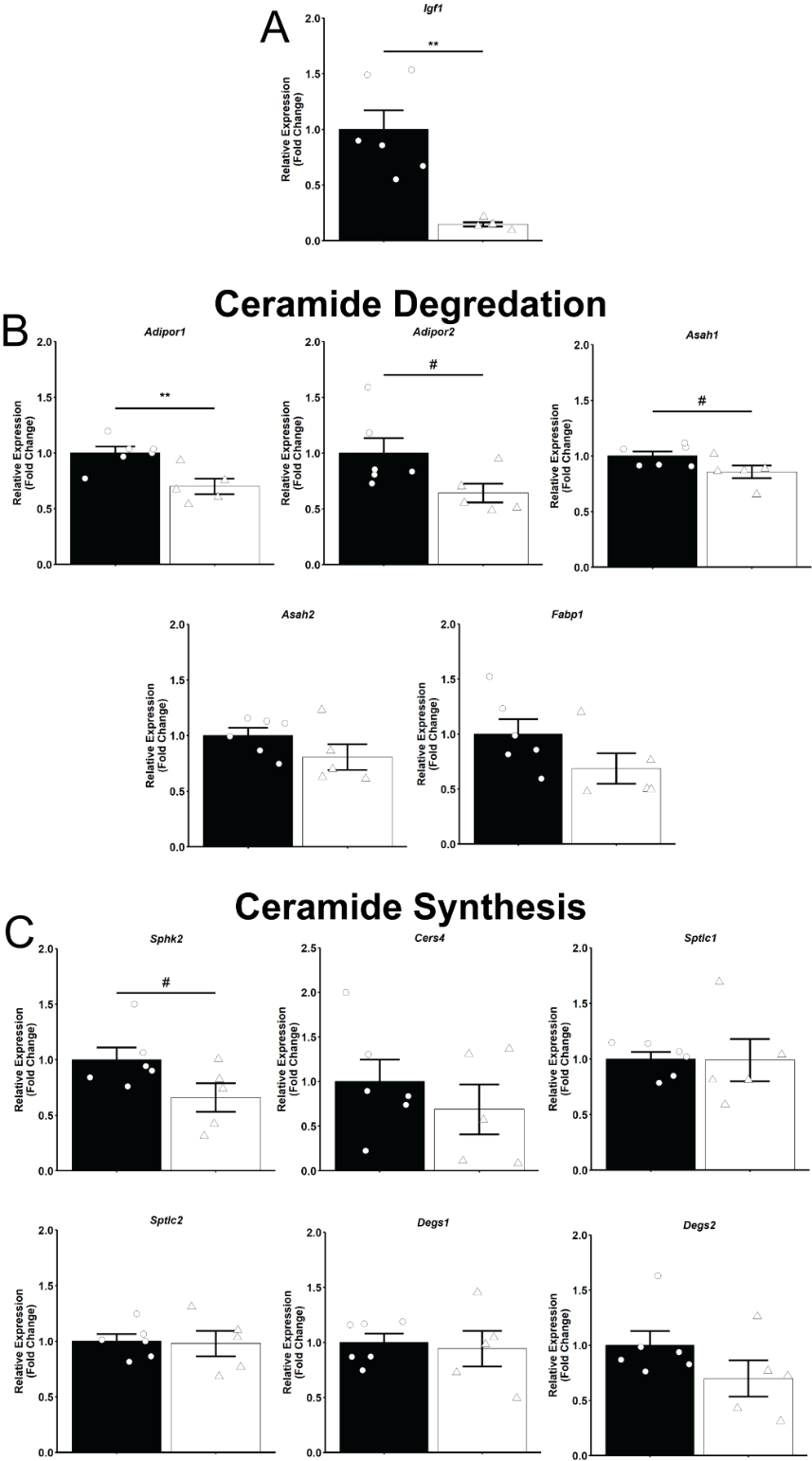


**Supplemental Figure S1**. Relative expression of *Igf1* (**A**), genes regulating ceramide degradation (**B**) and genes regulating ceramide synthesis (**C**) in the liver of 7mo GHRH-KO and WT females. #p<0.1; **p<0.01 as determined by two-tailed student’s t-test. Data presented as fold change relative to WT mice; bars show mean ± SEM with points representing individual mice. N=5-6 per group. *Actb* was used as an endogenous control.

**Supplemental Methods**

*Mice*

We employed mice with a CRISPR/Cas9 mediated deletion of growth hormone-releasing hormone (GHRH) as previously described (Icyuz, Fitch, Zhang, Challa, & Sun, 2020). These mice were maintained on a mixed BALB/cByJ X C57BL/6J genetic background to increase fecundity and minimize artifactual findings resulting from a homogenous genetic background. Mice were group housed at 4-7 individuals per cage in a pathogen-free facility maintained at 20-23 degrees C on a 12-h light and 12-h dark cycle. All mice had *ad-libitum* access to standard drinking water and rodent diet (NIH-31) except where mice were fasted. All mice used in this study were females between the ages of 3-15 months, as indicated in the results section or figure legends. All experimental protocols utilizing live animals were approved by the University of Alabama at Birmingham institutional animal care and use committee.

*Serum/Tissue Collection and Lipidomic Analysis*

Whole blood was collected from ambient-fed mice via cardiac puncture following carbon dioxide-induced anesthesia and was allowed to clot for 20 minutes at room temperature. Whole blood was separated by centrifugation (10 minutes at 3000x**g**, 4 degrees C) and the supernatant was collected and stored at -80 degrees C before analysis. Liver tissue was dissected, snap frozen on dry ice, and either processed for RNA as detailed below or analyzed for lipidomics.

Lipid extractions were conducted as we previously described (Poss et al., 2022) and the mass spectrometry methods have been detailed (Chaurasia et al., 2019; Poss et al., 2022). Briefly, extracts were resolved on an Acquity CSH C18 1.7 μm 2.1 × 50 mm column with a 1.7 μM VanGuard Pre-Column (Waters Corporation) maintained at 60°C and connected to an Agilent HiP 1290 Sampler and an Agilent 1290 Infinity Pump, equipped with an Agilent 1290 Flex Cube and an Agilent 6490 triple quadrupole (QqQ) mass spectrometer. Sphingolipids were detected using dynamic multiple reaction monitoring (dMRM) in positive ion mode. The source gas temperature was set to 210°C, with a gas (N_2_) flow of 11 L/min and a nebulizer pressure of 30 psi. The sheath gas temperature was 400°C, the sheath gas (N_2_) flow was 12 L/min, the capillary voltage was 4000 V, and the nozzle voltage was 500 V. The injection volume was 3 μL, and the samples were analyzed in a randomized order, with a pooled quality control sample injected 8 times throughout the sample queue. Lipids were quantified either against internal standards or, where standards were unavailable, based on HR-LC/MS, quasi-molecular ions and characteristic product ions as previously reported (Chaurasia et al., 2019; Poss et al., 2022). Lipid data was batch corrected using the Locally Weighted Scatter-plot Smoother (LOESS) algorithm (Rusilowicz, Dickinson, Charlton, O'Keefe, & Wilson, 2016).

*RNA extraction and RT-qPCR*

Mice were fasted overnight (16-18 h) to minimize potential effects of differential feeding states that can occur in group housed mice. Fasted females were sacrificed by carbon dioxide-induced anesthesia followed by cervical dislocation. The liver was dissected and RNA was extracted from liver tissue using TRIzol reagent (Invitrogen) following the manufacturer’s protocol. RNA preparations were treated with RNase-free DNase-I (New England Biolabs) according to the manufacturer protocol to remove any contaminating DNA. 1 µg RNA was reverse transcribed to cDNA using a high-capacity cDNA reverse transcription kit (Applied Biosystems) according to the manufacturer’s protocol. Real-time qPCR was carried out using Luna Universal qPCR Master Mix (New England Biolabs), a QuantStudio 3 thermal cycler (Applied Biosystems), and the appropriate primers (sequences provided in supplemental Table 1). Relative changes in gene expression were calculated using the 2^-ddCt^ calculation normalized to the wild-type group. *Actb* was used as an endogenous control.

*Statistical analysis*

The difference between two group means was compared using the two-tailed student’s t-test and the sequential goodness of fit correction (Carvajal-Rodríguez, de Uña-Alvarez, & Rolán-Alvarez, 2009) applied where multiple comparisons were made, with a p-value less than 0.05 being considered statistically significant. Principal component analysis was carried out using the *prcomp* function in the R programming language with default scale and center parameters applied and visualized using the *ggbiplot* function. Z-scores for heatmaps were calculated using the *scale* function in the R programming language with default parameters and visualized using the *pheatmap* function. Statistical analyses were carried out and figures were generated using the R programming language.

**References**

Carvajal-Rodríguez, A., de Uña-Alvarez, J., & Rolán-Alvarez, E. (2009). A new multitest correction (SGoF) that increases its statistical power when increasing the number of tests. *BMC Bioinformatics, 10*(1), 209. doi:10.1186/1471-2105-10-209

Chaurasia, B., Tippetts, T. S., Mayoral Monibas, R., Liu, J., Li, Y., Wang, L., . . . Summers, S. A. (2019). Targeting a ceramide double bond improves insulin resistance and hepatic steatosis. *Science, 365*(6451), 386-392. doi:10.1126/science.aav3722

Icyuz, M., Fitch, M., Zhang, F., Challa, A., & Sun, L. Y. (2020). Physiological and metabolic features of mice with CRISPR/Cas9-mediated loss-of-function in growth hormone-releasing hormone. *Aging (Albany NY), 12*(10), 9761-9780. doi:10.18632/aging.103242

Poss, A. M., Krick, B., Maschek, J. A., Haaland, B., Cox, J. E., Karra, P., . . . Summers, S. A. (2022). Following Roux-en-Y gastric bypass surgery, serum ceramides demarcate patients that will fail to achieve normoglycemia and diabetes remission. *Med, 3*(7), 452-467.e454. doi:10.1016/j.medj.2022.05.011

Rusilowicz, M., Dickinson, M., Charlton, A., O'Keefe, S., & Wilson, J. (2016). A batch correction method for liquid chromatography-mass spectrometry data that does not depend on quality control samples. *Metabolomics, 12*(3), 56. doi:10.1007/s11306-016-0972-2
